# Proton Content and Nature in Perovskite Ceramic Membranes for Medium Temperature Fuel Cells and Electrolysers

**DOI:** 10.3390/membranes2030493

**Published:** 2012-07-25

**Authors:** Philippe Colomban, Oumaya Zaafrani, Aneta Slodczyk

**Affiliations:** LADIR, UMR7075 CNRS, Université Pierre et Marie Curie (UPMC); 4 Place Jussieu, Paris 75005, France; Email: zaafrani@glvt-cnrs.fr (O.Z.); slodczyk_aneta@yahoo.com (A.S.)

**Keywords:** perovskite, proton conductor, ceramic, membrane, QNS, TGA, IR, Raman

## Abstract

Recent interest in environmentally friendly technology has promoted research on green house gas-free devices such as water steam electrolyzers, fuel cells and CO_2_/syngas converters. In such applications, proton conducting perovskite ceramics appear especially promising as electrolyte membranes. Prior to a successful industrial application, it is necessary to determine/understand their complex physical and chemical behavior, especially that related to proton incorporation mechanism, content and nature of bulk protonic species. Based on the results of quasi-elastic neutron scattering (QNS), thermogravimetric analysis (TGA), Raman and IR measurements we will show the complexity of the protonation process and the importance of differentiation between the protonic species adsorbed on a membrane surface and the bulk protons. The bulk proton content is very low, with a doping limit (~1–5 × 10^−3^ mole/mole), but sufficient to guarantee proton conduction below 600 °C. The bulk protons posses an ionic, covalent bond free nature and may occupy an interstitial site in the host perovskite structure.

## 1. Introduction

Considering the recent oil crisis and global warming problems, the hydrogen economy appears very promising. Hydrogen (produced by medium/high temperature electrolysis) can be oxidized in fuel cells to produce reversible electricity and/or can be used to convert CO_2_ into syngas [1,2,3,4,5,6,7]. The core of an electrolyzer can be built of a gas tight ionic conductor membrane: an oxygen vacancy conductor (stabilized zirconia or ceria [7,8,9,10]) or a proton conductor (perovskites [1,2,3,6,7,11,12,13]). The oxygen vacancy conductors work at high temperatures, usually above 800 °C. The proton conducting electrolytes operate between 400 °C and 600 °C and consequently appear as a more economic solution: The temperature values are high enough to avoid expensive noble catalyzers but low enough to optimize the costs of industrial devices. 

The high potential of perovskite ceramics as electrolytic membranes was shown in the 1980s, following the pioneer work of Forrat *et al*. [1] First devices such as water steam electrolyzers or gas separators were tested [11,12,13]. Since the presence of protons is not intrinsic to the host perovskite structure, a compound with a general formula of A^2+^B^4+^O^2−^_3_ first has to be modified with a few mol% trivalent cations—usually lanthanides (Ln) or Rare Earths (RE)—at the B site to form oxygen ion vacancies [3,4,7,14,15,16,17,18,19,20], namely A^2+^B^4+^_1−x_Ln^3+^_x_O^2−^_3−x/2_. Additionally, in order to incorporate the protons, such an oxygen-deficient structure should be annealed under water vapor pressure at medium to high temperatures [4,21,22,23,24]. 

One of the most important criteria to classify a perovskite ceramic as a good gas-tight electrolytic membrane is its high mechanical and chemical stability over thousands of hours under severe operating conditions: high temperature, electrical field, chemical gradient and vapor pressure cycling. Optimization of the materials and devices requires the identification of the protonic species, their nature, location in the hosting framework and the measurement of their short- and long-range dynamics. Consequently, different perovskite materials are widely investigated [1,2,3,7,11,12,13,14,15,16,17,18,19,20,21,22,23,24]. Despite intensive studies, there are however many problems which should be clarified prior to successful industrial application. The origin of these problems arises from the very complex physical and chemical behavior of the materials containing the protons. As pointed out in the introduction of the book “Proton conductors” [3], the very small size of a proton, in between that of the electron and the smallest “normal” Li^+^ ion, and the lack of electrons lead to protons being considered as unique species. Consequently, the physics and chemistry of protons—and of materials containing protons—are unique and can be named Protonics, a topic different from Electronics and Ionics. 

In this article, based on our previous studies on Ba/Sr-based zirconate ceramics [22,23,24,25,26,27,28], we will show that ignorance of fundamental aspects related to the proton type, such as stability *vs*. water pressure, surface or bulk moieties, as well as the proton content and nature, has important consequences: firstly on the understanding of perovskite proton conductor behavior, and secondly on the industrial applications as electrolytic membranes. 

## 2. Results and Discussion

### 2.1. Towards Understanding of the Protonation Process

**Figure 1:**
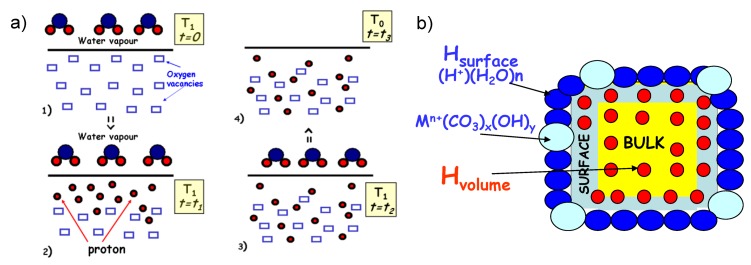
(**a**) Sketch of protonation process: (1) dissociation of water molecules at the surface of the oxygen-deficient perovskite; (2) filling of oxygen vacancies near the ceramic surface at temperature T_1_; a few days are required; (3) a diffusion of protons and V_O_/O^2−^ species through a ceramic is required to obtain homogeneous protonation; (4) stable, quenched state after protonation and cooling at T_o_ < T_1_; (**b**) Sketch of protonated ceramic: homogeneous distribution of bulk protonic species between a ceramic surface and bulk accompanied by the presence of protonic moieties adsorbed to the surface.

As already mentioned in the introduction, the presence of protons is not intrinsic to the perovskite structure. It is necessary to introduce/incorporate them, *i.e*., to protonate a material. In the protonation process, the water molecules, dissociated on the oxygen-deficient host perovskite structure (Figure 1a, Equation 1), should fill the oxygen vacancies and are the source of protons. Note that the dissociation mechanism has not been fully understood yet. 

                                      H_2_O => OH^−^ + H^+^ and/or H_2_O => O^2−^ + 2 H^+ ^                                     (1) 

**Figure 2:**
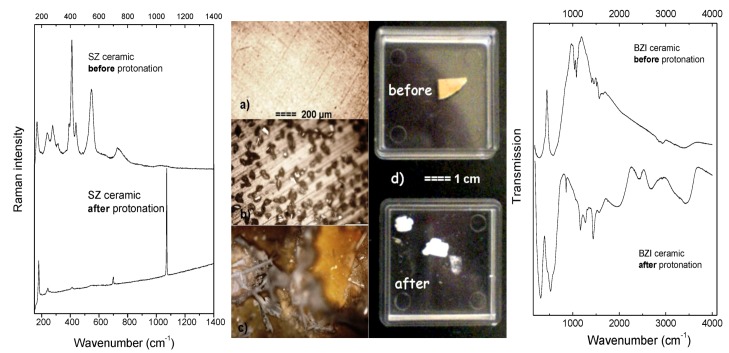
Surface of SZYb high dense ceramic (99%) (**a**) before; and (**b**) after protonation at 200 °C under 15 bar during 96 h; low temperature, low-pressure protonation conditions and the use of carbonate-containing water lead to the appearance of SrCO_3_ crystals. The Raman spectra characteristic of the ceramic surface before protonation—vibrational signature of perovskite structure, and after—vibrational signature of SrCO_3_ (Table 2) are given; (**c**) Surface of BZYb sample protonated during 23 days at 200 °C under 15 bar pH_2_O-important layer of carbonates and hydroxides is well seen [24]; (**d**) Comparison of the BZI sample [26] before protonation—ceramic piece, and after protonation—almost powder. Its low density and the presence of secondary phases lead to crumbling and chemical decomposition. The IR spectra characteristic of the ceramic surface before protonation—vibrational signature of perovskite structure, and after—vibrational signature of BaCO_3_/Ba(OH)_2_, nH_2_O (Table 2) are given.

Obviously, in the beginning of the protonation process, the mechanism takes place near the ceramic surface. Since after progressive filling of vacancies, the process may be slowed down, the protonation into the core/centre of the ceramic requires a diffusion of protons, V_O_ and O^2−^ species. Consequently, the choice of protonation conditions—temperature, water pressure and thermal treatment duration—is very important. The samples discussed in this article were protonated using high temperatures (200–600 °C) and high water vapor pressure (15–100 bars) in autoclaves during many days. The high water pressure allows the selection of stable ceramics. However, such a treatment during several days under 10–20 bar water pressure, rapidly crumbles poor and medium quality membranes (with reactive second phases at the grain boundary, see examples in Figure 2).

The aim of successful protonation is to obtain the homogeneous distribution of bulk protons—unique species responsible for the conduction across a membrane (Figure 1b). Simultaneously, it is necessary to avoid the absorption of protonic moieties on a material surface as much as possible, hindering the good H_2_/O_2_ conversion, in the form of secondary phases such as carbonates, hydrates, hydroxides, or more complex structures, *i.e*., complex second phases *i.e*., A(OH)_x_(CO_3_)_y_, nH_2_O [28]. 

Our study [22,23,24,25] revealed that the protonation process is very complex and strongly dependent on many parameters: (i) the used sample (crystallographic purity, substituting ion nature and level/oxygen vacancy content, alkali-earth element, *etc*.); (ii) the sample density/porosity (activity of surface); and (iii) the protonation conditions (temperature, water pressure, time, sample thickness). Successful protonation of a sample requires a compromise of all these parameters and in consequence is far from being trivial. For example, a high porosity promotes protonation by increasing the surface area where water dissociation takes place but simultaneously favors the adsorption of the protonic species at the sample surface. 

In order to go further into the comprehension of the protonation process we have protonated hundreds of perovskite ceramics of different composition, different densities and in different protonation conditions. Table 1 lists the most representative examples. Note that more important details can be found in [24]. It should be stressed that in order to determine the influence of the protonation process on a sample, each ceramic was carefully examined by Raman and IR spectroscopy before and after protonation (Figure 2). Since the secondary phases are often limited to traces and exhibit poor crystallinity, it is difficult to detect them using diffraction techniques. On the contrary, the presence of carbonates, hydroxides, hydrates, even if only in traces, gives rise to very characteristic IR and Raman peaks [28] and consequently allows an easy and efficient detection (Figure 2, Table 2). 

**Table 1:** Comparison of protonation conditions performed for different perovskite proton conductors: BaZr_0.25_In_0.75_O_3−__δ_ (BZI) [26], SrZr_0.9_Ln_0.1_O_3−__δ_ (SZYb) and BaZr_0.9_Ln_0.1_O_3−__δ_ (BZYb) [22,23,24,25].

Ceramic	BZI	SZYb	SZYb	SZYb	BZYb	BZYb
density	< 90%	~ 99%	~ 94%	~ 99%	~ 98%	~ 98%
Phase	Traces of 2nd phases	pure	pure	pure	pure	pure
Water type	decarbonated	decarbonated	decarbonated	Non-decarbonated	decarbonated	Non-decarbonated
Protonationconditions	300 °C	500 °C	200 °C	200 °C	250 °C	200 °C
	80 bar pH_2_O	80 bar pH_2_O	15 bar pH_2_O	15 bar pH_2_O	40 bar pH_2_O	15 bar pH_2_O
	96 h	96 h	96 h	200 h	72 h	550 h
Remark	Crumbling (Figure 2d)	Homogeneous surface-core proton distribution (Figure 5b)	IR spectrum (Figure 7b)	SrCO_3_ crystals on surface (Figure 2b)	Protons near surface (Figure 5a)	SrCO_3_ crystals and hydroxides on surface (Figure 2c)
		IR spectrum (Figure 7a)				
Proton content *Surface* & bulk	–	SrZr_0.9_Yb_0.1_O_3-__δ_ **H_0.005_**, *0.0008H_2_O*	SrZr_0.9_Yb_0.1_O_3-__δ_ **H_0.002_**_,_ *005H_2_O*	–	–	–

**Table 2:** Main Raman and IR signatures characteristic of Sr-based carbonates, hydroxides and hydrates. The strongest peaks are marked in bold. The so-called ABC bands characteristic of strongly H-bonded species are marked in italic. Note, since in real conditions the second phases are present in a complex form Sr(OH)_x_(CO_3_)_y_, nH_2_O, more or less hydrated, coexistence of peaks and wave number shifts are usually observed [25,26,28].

Compound	Raman (R) and IR vibrational signature (cm^−1^)		
	Lattice & deformation modes	νCO_3_ modes	νOH modes
SrCO_3_	R 106, **148**, 181, 244, 697	**1071**, 1450	–
	IR 700, 857	1071, **1480**	–
Sr(OH)_2_	R	–	**3592**, **3606**
	IR	–	3592, 3606
Sr(OH)_2_ 0.3H_2_O	R	–	**3488**, **3500**, 3592, 3618
	IR	–	*1710*,*2345*,***2940***, 3500, 3592
Sr(OH)_2_ 1H_2_O	R	–	3297, **3488**, **3594**, 3610
	IR	–	***1710***,*2370*,***3005***, 3297, 3398, 3488, 3592

Some important conclusions concerning the protonation process can be found: (i) The lower the ceramic densification (the higher the active surface area), the higher the absorption of surface protonic moieties; (ii) according to Ellingham-Richardson diagrams [29], the protonation at low temperatures (~<200 °C) and the use of carbonate-containing water enhance the adsorption of surface protonic species (Figure 2b,c); (iii) the presence of secondary reactive phases in a pristine sample leads to crumbling (Figure 2d) and chemical decomposition/hydrolysis [26]; (iv) high temperature limits the carbonation [29]; (v) high temperature and high water vapor pressure conditions enhance the homogeneous distribution of bulk protons [24,25]; (vi) typically, an homogeneous, bulk-proton saturated state requires about 100 hours of protonation in the case of 1 mm thick ceramic (static conditions) [23]. 

**Figure 3:**
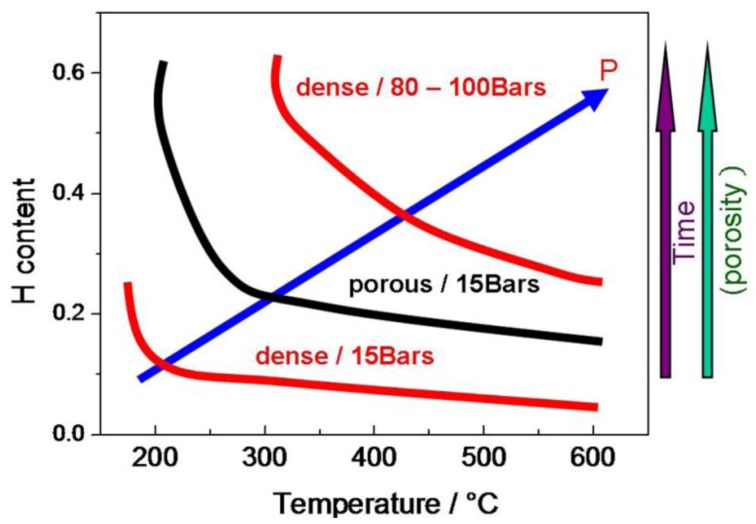
Schematic summarizing the H bulk content as a function of protonation conditions (temperature, water pressure) and sample parameters (densification, *i.e*., the active surface area/porosity).

The effect of protonation conditions on bulk proton insertion is summarized in Figure 3. It should also be stressed that the “extreme” treatment under high pressure at high temperatures allows us to rapidly select the most chemically stable ceramic membranes. 

The protonation process has not been fully understood yet and hence is not well optimized. Consequently, a qualitative and quantitative control of protonation is necessary to distinguish between the bulk and surface protonic species.

### 2.2. The Content and Distribution of Bulk Protons

It is extremely surprising that the bulk proton content is systematically not determined in literature. The proton content is mostly postulated based on the oxygen vacancy content estimated due to the RE/Ln^3+^ substitution. Note that because of the charge equilibrium requirement, 1 mole of substituting ions can create ½ mole of oxygen vacancy. Moreover, the differentiation between the bulk protons and protonic moieties adsorbed on a material surface is systematically ignored as well. How is it then possible to determine the complex behavior of a perovskite electrolytic membrane without knowing its exact composition and eventual pollutions?

Actually, the precise proton content determination is difficult. Direct and indirect methods can be distinguished. Since the incoherent cross-section of hydrogen/proton element is huge (^H^σ_inc_ = 80.26 barns) in comparison with any other element constituting the perovskite structure, (for example σ_inc_ ~0.001 for O and σ_inc_ ~0.02 for Zr), neutron scattering experiments (elastic, quasi-elastic neutron scattering (QNS), inelastic neutron scattering (INS) and neutron diffraction) are among the best direct methods allowing the measurement of small hydrogen contents [4,21,22,27,30,31,32]. It should be stressed however, that in the case of proton conducting materials the neutron techniques are rarely used to measure the proton content (Table 3) [22,25,33,34]. The techniques based on nuclear reaction between an ion beam and protons, for example Rutherford backscattering spectrometry (RBS), elastic recoil detection (ERD) or elastic recoil coincidence spectroscopy (ERCS), can also offer tools to measure the proton content, but to date their use is very limited [35,36,37,38]. Within the indirect methods, the thermal ones, especially thermogravimetric analysis (TGA) are mostly used. Note however that the TGA does not allow determining the H content as directly as the neutron measurements. Namely, the total weight loss observed during the temperature increase can reveal the departure of free molecules of water, chemically bound water below 300 °C and protonic entities such as OH^−^ and/or H^+^ [18,19,22,23,24,25,28].

**Table 3:** Techniques offering proton content determination in proton conductors.

Technique	Sample	Reference
QNS (quasielastic neutron scattering)	SrCe_0.95_Yb_0.05_O_2.985_	[33,34]
	BaZr_0.9_Yb_0.1_O_2.995_	[22]
	SrZr_0.9_Yb_0.1_O_2.995_	[25]
RBS(Rutherford backscattering spectrometry)	beta alumina	[35]
ERD (elastic recoil detection)	BaCe_1−x_Y_x_O_3−δ_	[36]
	SrCe_1−x_Y_x_O_3−δ_	
ERCS (elastic recoil coincidence spectroscopy)	BaCe_0.9_Y_0.1_O_3−δ_	[37]

**Figure 4:**
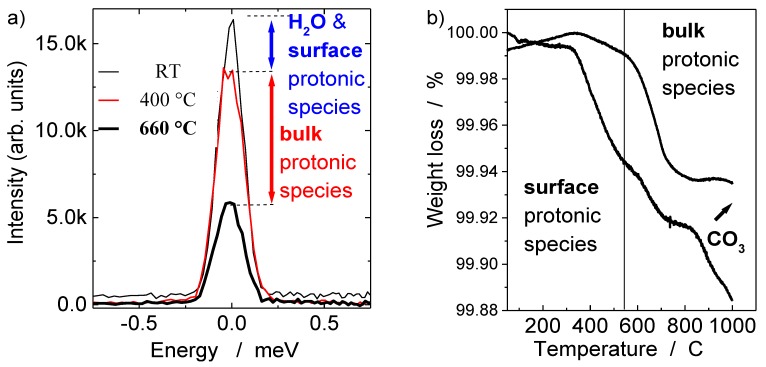
(**a**) Elastic neutron spectra measured at RT, 400 °C and 660 °C under high vacuum using time-of-flight spectrometer; (**b**) thermogravimetric (TG) curves recorded for the ~ 94% dense ceramic and the very high dense (~99%) ceramic (He atmosphere). Samples were preliminarily dried at 300 °C to eliminate traces of water molecules adsorbed on the ceramic surface.

In order to measure both the bulk proton and surface moieties contents we profited of the advantages of direct (QNS) and indirect (TGA) methods (Figure 4a,b). 

#### 2.2.1. Bulk Proton Content by Quasi-Elastic Neutron Scattering

Figure 4a compares the intensity of QNS peaks recorded at a few characteristic temperatures for highly dense (99% of theoretical density) Ln-modified SrZrO_3−δ_ ceramics using a Time-of-Flight spectrometer. The elastic peak intensity variations are directly proportional to the hydrogen content, according to the following formula (Equation 2):

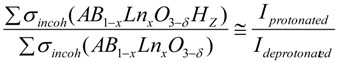
(2)
where *σ_incoh_* is the incoherent neutron cross sections; and *I* the elastic peak intensity for protonated and deprotonated (or non-protonated) compounds, respectively. 

As it can be seen in Figure 4a, the highest intensity is detected at room temperature in agreement with the presence of free water adsorbed on the ceramic surface as well as the surface (traces of hydrocarbonates, hydroxides, *etc*.) and bulk protonic species. Heating above 300 °C, especially under dynamically high vacuum conditions, allowed the ceramic surface to be cleaned from the surface protonic species [24,25,27]. A comparison of the intensity values recorded at RT and 400 °C clearly shows that in the case of very high density ceramics, the contribution of surface protonic species is low, much lower than the content of bulk protonic species. The decrease of the intensity detected above 600 °C reveals the deprotonation process, *i.e*., the departure of protons. The comparison of intensity values measured at 400 °C and 660 °C allows the calculation of the content of bulk hydrogen species. Note that the peak observed at 660 °C is an elastic contribution of the host perovskite lattice. The bulk proton content is low, *i.e*., ~0.005 mole/mole. Hempelmann *et al*. [33,34] determined that the proton content in cerate ceramic is 10 times higher, *i.e*., ~0.02. The difference can be explained first of all by the different nature of the sample, *i.e*., strontium cerate and its lower density (~95%). However, one may ask a question: Did Hempelmann *et al.* [33,34] only measure the bulk protonic species or both the bulk and surface protonic moieties? 

#### 2.2.2. Bulk Proton Content by Thermogravimetric Analysis

The bulk proton content determined with the neutron technique is consistent with the results of TGA measurements. Note that the TG analysis is performed in a He atmosphere using a Pt crucible in order to enhance the accuracy of detecting very low mass losses in the temperature range where heat transfer is controlled by gas convection. Figure 4b compares the TGA curve characteristics of the high density (99%) and the lower density (94%) Ln-modified SrZrO_3−δ_ ceramics. As it can be seen in Table 1 both ceramics were protonated in different conditions in order to determine the influence of the protonation method on the bulk and surface protonic species contents. Note that both samples were preliminarily dried at 300 °C in order to eliminate the contribution of free surface water. Two or three characteristic mass losses can be detected as a function of the sample. The first one, detected near 400 °C can be attributed to the departure of the surface protonic species (higher departure temperature, higher chemically bonded (acidic) water); the second one—above 600 °C—is related to the elimination of the bulk conducting species whereas the third one observed above 800 °C, characteristic of ceramics containing the carbonates only, corresponds to carbonates decomposition [28]. As we can see, the 94% density sample possesses higher values of surface protonic species in relation with its higher active surface area. The very high density sample shows traces of surface protonic species only in good agreement with neutron measurements (Figure 4a). The concentrations of bulk and surface protonic species for both samples are given in Table 1. 

#### 2.2.3. Distribution of Protonic Species: Raman Profilometry and Neutronography

**Figure 5:**
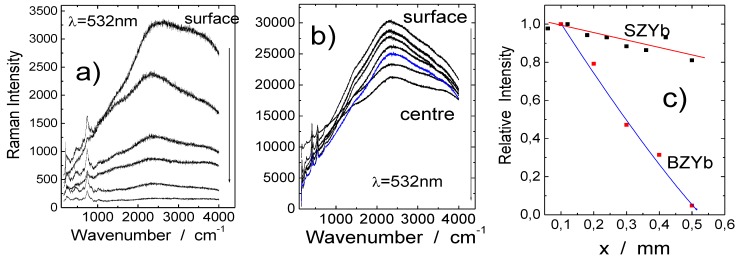
Raman profilometry method: Spectra with characteristic backgrounds of (**a**) Ln-modified BaZrO_3−δ_ (protonation at 250 °C under 40 bar during 72 h-protons near surface only); (**b**) Ln-modified SrZrO_3−δ_ (protonation at 500 °C under 80 bar during 96 h homogeneous distributions of protons) were collected along the ceramic section, from the surface to the centre, just after fracture [23]; (**c**) The distributions of the spectroscopic signal measured across the half section (sample thickness = 1 mm) follows Fick’s law.

Another important aspect is the proton distribution in a ceramic. The homogeneity of the proton distribution can be followed using the so-called *Raman profilometry* method [23]. This method is based on a careful analysis of an intense background, centered near 2,500 cm^−1^, assigned to electronic defects associated with protons inserted at temperatures below ~300 °C, which can be detected in the Raman spectrum [23,24]. The comparison of the Raman study of zirconate and titanate perovskites revealed that the broad Raman background intensity is proportional to the content of the protonic species as well as roughly to the conductivity of the material. Figure 5a,b show the Raman spectra with characteristic broad backgrounds recorded on the section—from surface to its center—of BaZrO_3_ and SrZrO_3_ ceramics (see Table 1) protonated in different conditions. The background intensity as the function of the position along the sample section (Figure 5c) reflects the profile expected from Fick’s diffusion laws [23]. As it can be clearly seen, the curves exhibit different forms. The first one, characteristic of Ln-modified BaZrO_3_ ceramic (Figure 5a), reveals that the protonic species are present at/near the ceramic surface only. In the lack of easy diffusion of the O^2−^/V_O_ species requiring high temperature, it is highly probable that the filling of vacancies is limited to the near-surface layers only. On the contrary, the curve presented in Figure 5b, characteristic of Ln-modified SrZrO_3_, clearly shows a very homogeneous distribution of the protonic species in the sample, which confirms a successful protonation. 

**Figure 6:**
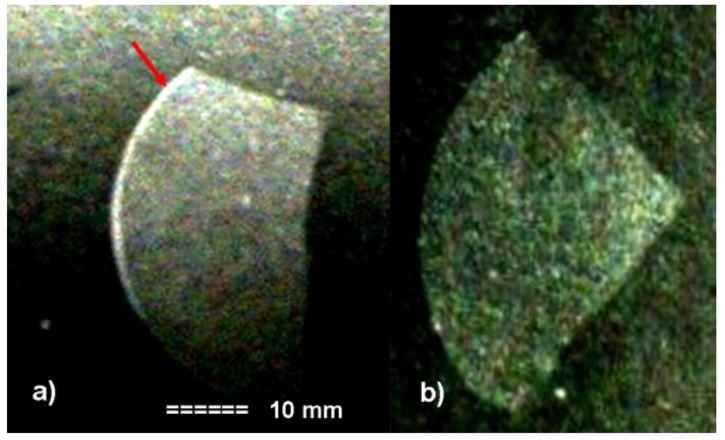
Neutronography micrographs characteristic of two Ln-modified SrZrO_3−δ_ceramics. Hydrogen-rich regions appear in white. (**a**) On the ceramic surface (250 °C, 40 bar, 72 h); (**b**) throughout the ceramic (310 °C, 90 bar, 96 h).

Since the neutronography method is a suitable tool for the detection of hydrogen elements in a dense material matrix, we performed the first tests on high dense perovskite ceramics containing the protonic species. The results of neutronography tests performed at Léon Brillouin Laboratory (Saclay, France) using radiographic films as detector on two Ln-modified SrZrO_3−δ_ ceramics are presented in Figure 6. The presence of protonic species is detected as white contrast. As it can be clearly seen the ceramic presented in Figure 6a contains protonic species mainly on the surface whereas in the case of Figure 6b the protons seem to be distributed throughout the ceramic.

### 2.3. Bulk and Surface Protonic Species—Importance of Differentiation

Since it is almost impossible to avoid the presence of surface protonic species, it is extremely important to perform the differentiation prior to any measurement. As we have shown in the previous paragraph, the amount of surface protonic moieties (undesirable secondary phases) can be particularly enhanced if materials with an important active surface are used, *i.e*., powders or poorly densified ceramics [25,26,28]. In consequence, the quantity of surface protonic moieties may be comparable or even more important than the bulk proton content. 

#### 2.3.1. Proton Conductivity

The ignorance of the differentiation between bulk and surface protonic species can have serious consequences. First of all, the important presence of protonic species adsorbed on a material’s surface may significantly distort experimental data. Conductivity measurements can be cited as a good example. The surface secondary phases, such as hydroxides, especially in liquid-hydrolyzed form, are very good proton conductors. Jalarvo *et al*. [39] showed that in the case of Sr_4_(Sr_2_Nb_2_)O_11_·nH_2_O and Sr_4_(Sr_2_Ta_2_)O_11_·nH_2_O proton conducting perovskite, the secondary phases such as Sr(OH)_2_ contribute to and even dominate the proton conduction. In consequence, the Sr content in the pristine perovskite material is strongly decreased, which leads to a deterioration of the sample. IR analysis of similar compounds show strong ca. 1,450 cm^−1^ band characteristic of carbonated surface species [40,41].

Our study revealed that the conductivity of a very dense ceramic, measured directly in a pressurized water steam electrolyzer, achieves 10^−2^ S/cm below 600 °C and its value is stable during hundreds of hours [22,25]. On the contrary, low dense ceramics with a high level of surface protonic species have a very short lifetime under high H_2_O pressure. Namely, the formation of OH^−^ species leads to the loss of mechanical properties or even to ceramic crumbling and structural decomposition in the case of highly substituted compounds [26].

#### 2.3.2. Proton Site

The analysis/interpretation of X-ray and/or neutron diffraction experiments also suffers from the presence of surface protonic moieties. Despite intensive studies [42,43,44,45,46,47], the exact proton localization in the host perovskite structure has not been definitively determined yet. Indeed, an extremely high incoherent cross section together with an extremely low coherent cross section makes the proton site determination a big challenge even at low temperatures far from conduction conditions. The analysis of some attempts, however, suggests the source of these difficulties. First of all, the sample composition is estimated. Namely, the proton/deuteron content is fixed due to the estimation based on the substituting atom and expected oxygen vacancy contents. Consequently, the obtained reliability factors question the results of Rietveld refinement [48]. Moreover, most of the authors performed diffraction studies on powders or poorly densified ceramics at room or at very low temperatures [42,43,44,45,46,47], which significantly enhances the probability of the presence of surface protonic species. The presence of some secondary phases, mostly hydroxides, was evidenced even by diffraction [44,45]; note that if the second phases are limited to traces only, their detection is impossible in diffraction experiments [26,28]. 

#### 2.3.3. What Is The True Nature of Bulk Proton?

**Figure 7:**
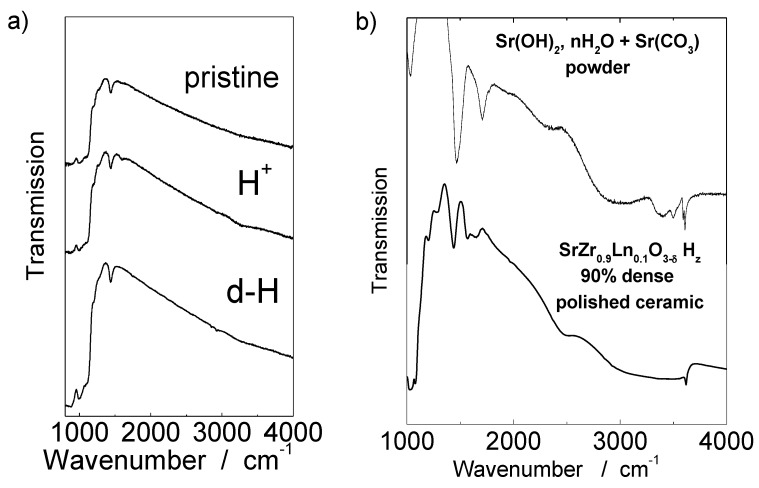
(**a**) IR spectra recorded on highly dense (99%) polished, thin (~120 µm) pristine (non-protonated), protonated and deprotonated Ln-modified SrZrO_3−δ_ ceramics; (**b**) Comparison of IR spectra characteristic of low dense (94%) polished, thin Ln-modified SrZrO_3−δ_ ceramic with a Sr(OH)_x_(CO_3_)_y_, nH_2_O powder dispersed in CsI matrix (see Table 2 for band wave numbers and assignments).

The lack of differentiation between the bulk and surface protonic moieties also makes the true proton nature very ambiguous and hinders a clear analysis of QNS data. Recently, most of literature data has “accepted” the previous statement that protons in perovskite penetrate the oxygen covalence sphere to create OH groups. This thesis is based on the presence of important IR signatures detected in the OH-stretching region, *i.e*., 1,500–4,000 cm^−1^ [49,50,51,52,53]. Note that such a rich IR signature is also considered to be undisputable proof of a successful sample protonation. The proton conduction is described according to the Grotthuss mechanism [49]. However, our results obtained on highly dense polished ceramics, which according to neutron and TGA analyses contain mostly bulk protons, allowed us to question the hydroxyl nature of protons. As presented in Figure 7a, the IR spectrum characteristics of highly dense SrZrO_3_ does not show any signature in the O-H-stretching region, similarly to samples in non-protonated and deprotonated states. IR spectra recorded on optical clear glass pieces with similar thickness show characteristic hydroxyl ion and water signatures [54]. For clarity, Table 2 lists vibrational signatures of Sr-based carbonates, hydrates and hydroxides. The lack of specific signature on protonated polished ceramic confirms the absence of hydroxyl species. This reveals that the true bulk proton does not give any IR signature, which can be explained by the presence of another type of hydrogen species. The most reasonable hypothesis is interstitial protons, free of any specific strong hydrogen bonding. This type of proton was detected for the first time in 1997 by inelastic neutron scattering in some complex oxides and is called the ionic proton [21,55]. Another type of non-covalent bonded protons that do not show IR signature is a proton gas as observed in polyaniline by neutron scattering [21,56].

In order to go further into the comprehension of this discrepancy we simultaneously performed the IR study on low dense ceramics protonated in conditions favoring the adsorption of surface moieties. The low density sample—see Figure 7b—exhibits a significant IR signature in the 1,400–4,000 cm^−1^ region (Table 2): A very broad structured massif similar to that recorded for well characterized materials hosting M^n+^(H_2_O)_n_ species [3,28] and a very thin peak located near ~3,600 cm^−1^ assigned to the OH^−^ vibrator [28]. Such a IR spectrum is very similar to that characteristic of Sr(OH)_x_(CO_3_)_y_ nH_2_O powder.

It should be stressed that most of the IR spectra of different proton conductors presented in the literature [49,50,51,52,53] are very similar to that typical of low density ceramic and powder. This indicates that the authors analyzed mainly, or even exclusively, the IR signatures of second complex phases instead of those of conducting protons. The description of their samples (powder or poor sintered ceramics) confirms the presence of a very important active surface. 

## 3. Experimental Section

Ln-modified SrZrO_3−δ_ and Ln-modified SrZrO_3−δ_ ceramic pellets with controlled densification (90%–99% of the theoretical density) have been elaborated by chemical route and sintered over 1,550 °C [22,23,24]. The BaZr_0.25_In_0.75_O_3−__δ_ ceramics (density < 90%) were prepared by solid-state reaction and sintered over 1,420 °C [26]. The crystallographic purity of pristine samples was controlled by X-ray/neutron diffraction, Raman scattering and IR transmission. The protonation process was performed at a high temperature (200–600 °C), high water vapor pressure (15–100 °C) in instrumented home-designed autoclaves during a few days. See Table 1 for more details. 

The H content was measured by elastic neutron scattering using 3-axes (1T1 and 4F1) and time-of-flight (Mibemol) spectrometers (Leon Brillouin Laboratory, Gif-sur-Yvette Cedex, France [25]). 

TG analysis used a Setaram Setsys instrument in the 25–1,000 °C temperature range, with a heating rate of 5 °C/min. A Pt crucible and He atmosphere were used to maximize the heat transfer and hence the measurement accuracy.

Raman spectra prior to the Raman profilometry were recorded using Infinity Dilor and/or HR Horiba Raman microspectrometers. A few wavelengths were tested (458 nm, 514 nm, 532 nm and 632 nm). 

IR transmission spectra were recorded in air on diamond optically polished ceramics (thickness 100–150 µm) before and after protonation in the 1,000–8,000 cm^−1^ range using FT Equinox 55 Irscope Bruker Optics microspectrometer. IR spectra of Sr-based hydroxide powder were recorded on a double beam IR spectrometer PE 983 under dry air flux using the CsI pellet technique [28].

## 4. Conclusions

Surprisingly, the exact hydrogen content is widely ignored in the literature. Most of the literature data are interpreted based on a postulated composition: The proton content is estimated due to the Ln/Re substituting ion content and the oxygen vacancy content. We pointed out that the bulk proton content is very low, only 10% of oxygen vacancies are filled by bulk protons. Such proton doping is however sufficient to guarantee significant proton conduction (>10^−3^ S/cm). The results of our (quasi-)elastic neutron scattering, TGA, Raman and IR studies clearly show the importance of differentiation between the bulk protons and the protonic moieties adsorbed on a material surface. This is related to the high complexity of the protonation process. Namely, the homogeneous distribution of bulk protons throughout a material without significant adsorption of the surface moieties requires the compromise between specific conditions of protonation (temperature, pressure, duration) and material properties (density, active surface area, compositions: Ln and oxygen vacancy contents). It is impossible to avoid the surface moieties, especially when the protonation is performed on powder or low density ceramic samples under ambient conditions. However, their content should be limited to traces, much lower than that characteristic of bulk protons. Consequently, the content of bulk protons and of eventual surface pollution should be controlled prior to any physical/chemical measurement. Note that by using a highly dense Sr-based zirconate ceramic as an electrolytic membrane of water steam electrolyzer, we established a hydrogen production [57]. This conducting bulk proton does not show any IR signature in the OH stretching region in the contrary to most of the data found in the literature. Consequently its nature cannot be described as an OH ion according to the previous theory. The lack of a IR signature reveals that this proton does not create a covalent bond with its environment and can be then considered as an ionic species occupying an interstitial site or delocalized along a conducting pathway (proton gas). This suggests that data in the previous literature are polluted by undesirable protonic moieties in the form of second phases such as hydroxides, hydrates, *etc*. These secondary phases are “unfortunately” very good proton conductors—they melt at ~400–600 °C—and hence can significantly enhance the conductivity carried out on perovskite proton conductors. 

To summarize, the behavior of protons and perovskite proton conductors is far from being understood but has to be well determined prior to successful industrial application within hydrogen economical devices. 
